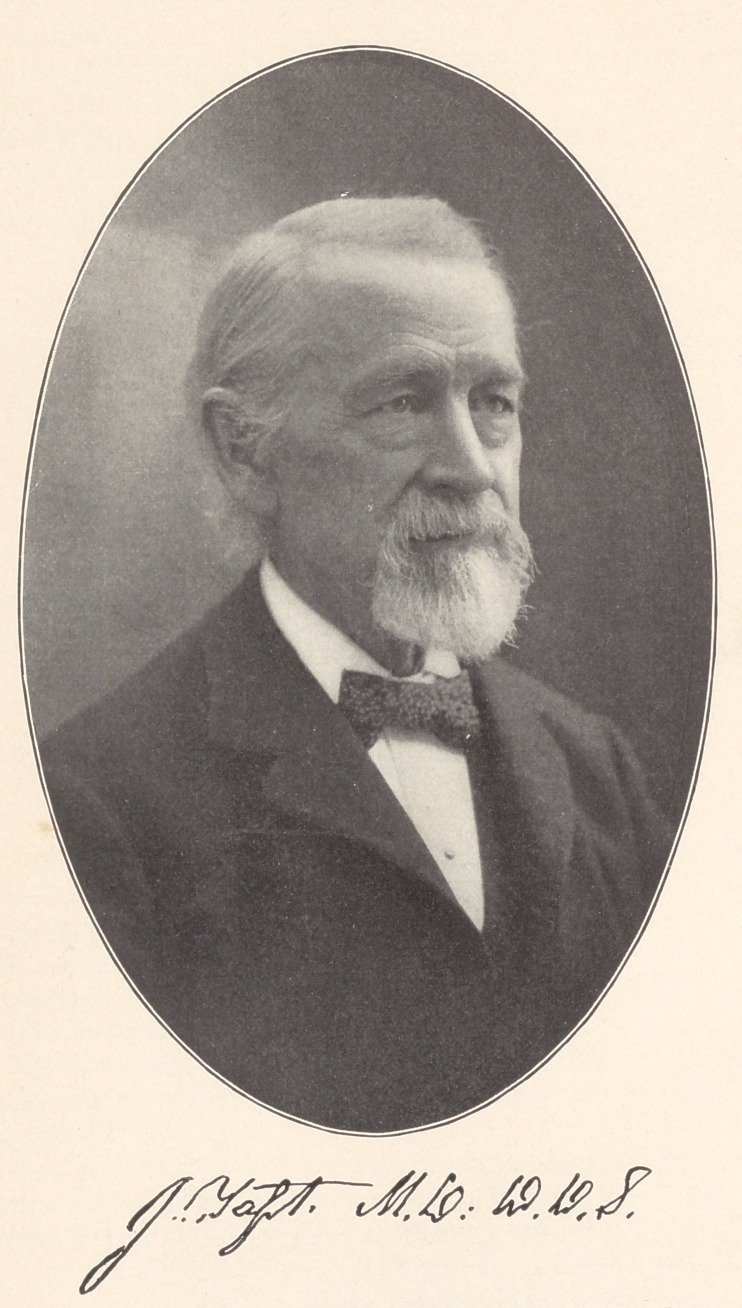# Jonathan Taft, M.D., D.D.S.

**Published:** 1903-12

**Authors:** 


### Obituary.

### JONATHAN TAFT, M.D., D.D.S.

Dr. Taft died at Ann Arbor, of apoplexy, at midnight, October 15, 1903.

This is the brief record of the last hours of one of the brilliant and most faithful men dentistry has known for more than half a century. He has been so much in the full glare of professional and educational life during all that period that the writers of his personal history seem to have been at a loss to mark any special work as peculiarly his own, for he has been part of everything that meant an advance in dental work. Yet, as stated in an editorial in the November number, he was not an original investigator in the sense that that word is generally understood.

Jonathan Taft was born September 17, 1820, in Russellville, Brown County, Ohio. His father, Lyman Taft, was a native of Massachusetts, removing to Ohio in 1818. His son received his education in the public schools and in an academy in Brown County. After graduating he taught school for several years. He began the study of dentistry with Dr. George D. Tetor, of Ripley, Ohio, in 1841, and began practice in that town in 1843. He removed to Xenia, Ohio, in 1844, and practised there until 1858.

He graduated from the Ohio College of Dental Surgery in 1850, and was appointed to the chair of Operative Dentistry and Dental Hygiene in 1854 in this college, and continued to perform the duties of this position until 1879. During most of the years he filled the important and responsible position of Dean of the College, and continued to perform its duties until, in 1879, he was called to take charge of the Department of Dentistry connected with the University of Michigan. It was in this institution that the best work of his life was accomplished. While the labor of organizing this department was shared by an able corps of teachers, it was due to his fame, national and international, that the school became, in a comparatively short period, recognized as one of the leading dental colleges of this country. For a considerable period it and the Department of Harvard University were the only dental colleges recognized in England. The school under his guidance continually maintained a high standard and it was the first to demand a four years' course of its matriculants, and that in advance of the decision of the National Association of Dental Faculties, making this period obligatory on all schools in its membership.

Dr. Taft remained at the head of this department until the present year, when he was retired, it is said, upon a pension. This dismissal has caused much bitter feeling among the alumni and the general public in Michigan, but the facts are not at hand to form any opinion as to the merits of the controversy. It remains, however, a matter of deep regret that he was not continued in his position as long as he was able to serve intelligently. This, it seems to the writer, was his due as a recompense for the great work he performed in building up the department.

In 1856 Dr. Taft became editor of the *Dental Register,* a monthly periodical devoted to the interests of the dental profession, and he closed his editorial labors in 1900, a period of forty-four years. This in itself was a record of faithful service not equalled by any one of his contemporaries, or is it at all probable that it will be imitated in the very near future. His labor upon this journal means a record of the modern life of dentistry in this country, for in that period it has developed from a crude mechanical calling into a near approach to a cultured profession.

In 1857 Dr. Taft became a resident of Cincinnati, establishing there a large and remunerative practice, and continued there until 1901, a period of thirty-three years.

In 1879 he resigned from the Ohio College of Dental Surgery and confined his educational work entirely to the Department of Dentistry of Michigan University, changing his place of residence from Cincinnati to Ann Arbor in 1901.

Dr. Taft has been a member of not only the local organizations of dentistry, but of all the national bodies organized during his life. He was made secretary of the American Dental Association at its organization at Niagara Falls in 1859, and in 1869 he was elected president of that body.

He was one of the original organizers of the National Association of Dental Faculties, at its first meeting in New York in 1884, and subsequently was elected to the presidency of that Association.

He was also an active member of the Institute of Dental Pedagogics. When the American Dental Association combined with the Southern Dental Association at Old Point Comfort, in 1897, Dr. Taft was present, and although it grieved him, as it did others, to part with the name and history of this organization, he felt it was for the good of dentistry, as a whole, that the sacrifice should be made, and, with the courage with which he was able to meet all changes, he entered into the new work of reorganization with the energy that had always been an inspiration to his colleagues.

His last active co-operation in the two national bodies—the National Association of Dental Faculties and the National Dental Association—was at the last meetings held at Asheville, N. C., in August last. To all appearances our old friend seemed, as he had been for years, active and alert to all that interested the younger generation.

In 1859 Dr. Taft prepared the work known as "Taft's Operative Dentistry." This was the first attempt to confine a book to the consideration of the subject of filling teeth and the pathological conditions connected therewith. Harris and other authors had preceded him, but they had combined mechanical dentistry with that of operative. It was the beginning of specializations in dentistry which have continued to the present time, increasingly dividing up the calling into a number of distinct but related vocations.

This work of Dr. Taft ran through several editions, and for some years was held as an authority and recognized as a text-book in all the colleges of the country. It was, unfortunately, not kept up to the standard of progress, and was eventually superseded by the works of younger men; yet, notwithstanding this, it must be regarded as one of the most important agencies in the general uplift of dentistry, and brought to its author a national as well as an international reputation.

Dr. Taft, as already stated, was not an originator. He must be considered in the light of an expounder of other men's work. His reports on various subjects at the national conventions, were models of clear statement, and his remarks in discussions always exhibited a grasp of the subject that insured him an attentive audience.

While holding positive opinions, he was not a controversialist in the sense of rousing bitter antagonisms, and yet he had the courage of his convictions and dared to carry these out in the face of much prejudice. When the writer advocated the admission of women into dentistry through a resolution offered at the American Dental Association held at Saratoga in 1869, he was not supported by any one, yet Dr. Taft very shortly thereafter introduced coeducation into his school and continued it until the day of his retirement. He evidently did not care to enter into a wordy contest on this subject, preferring to give results to the world, and these have, in combination with similar efforts in other schools, been entirely satisfactory, opening up a new field of industry to womankind the world over.

To the students under his care he was a professional father, sympathizing with them in their difficulties, and an able adviser in times of need. One of his grateful students, in a private letter, writes: He was one "whose hands have stayed and aided many students in a downward career. Few men know the work, in that direction, that Taft did. Money he used, and it was freely given." The fact that the alumni of his school have always been his devoted friends is the highest honor that can be paid any educator. They, as students, could measure his qualities, and the verdict they have universally given shows unmistakably that the man was deservedly honored for his ennobling qualities.

Dr. Taft was religious without a taint of bigotry. He never made his religion a standard for other men to work up to and adopt. He was altogether too cosmopolitan a character for any narrow faith. He could cordially associate with men of all creeds, ever apparently mindful that all nations, kindreds, tongues, and peoples must reach the highest through many avenues and through many standards of faith.

One of the marked evidences of the power possessed by Dr. Taft over his professional associates is the fact that, notwithstanding the number of strong men during the period about 1850, he commanded respect and had thus early secured a wide reputation. The writer cannot recall a period when Dr. Taft's name was not familiar to him as one of the prominent and progressive men in dentistry.

The following quotation from an address delivered, as president, before the American Dental Association in 1869 furnishes the key to Dr. Taft's life-work. He stated then, "I have no sympathy or patience with the professional brother who, reposing in his quiet selfishness, or reclining upon his dignity, refuses to take part in the great labor of the day. The man who does not feel and yield to the impulses of the age—aye, is not fired with its spirit—belongs to by-gone days; by some mishaps his coming has been delayed a few generations."

The calling of dentistry has lost one of its most earnest men. His whole life has been devoted to elevating his profession. He has set an example that all may follow,—all should follow,—with profit to themselves and their fellows. The thing that Dr. Taft most fervently believed in was that which the dentists of to-day need the most,—a true professional spirit. If his death can bring the great body to a realization of the fact that this spirit is almost entirely lacking, his life will have been a blessing and his death will not have been in vain; for it has emphasized this fact in his life more prominently than any other of his many good and noble traits of character.

In 1842 Dr Taft was married to Hannah Collins, of Ripley, Ohio, who died in 1888, and in 1889 he married Miss Mary Sabine, of Cincinnati, who survives him. Two sons, Dr. Wm. Taft, of Brewster, N. Y., and Dr. Alphonse Taft, of Cincinnati, and one daughter, Mrs. A. T. Edwards, by the first marriage, are now living.

Funeral services were held at his home, October 17, and the body was removed to Spring Grove Cemetery, Cincinnati, for burial.

## Figures and Tables

**Figure f1:**